# Efficacy of Various Intracanal Medicaments in Human Primary Teeth with Necrotic Pulp against *Candida* Biofilms: An *in vivo* Study

**DOI:** 10.5005/jp-journals-10005-1406

**Published:** 2017-02-27

**Authors:** Jophie V Paikkatt, Sheela Sreedharan, Beena Philomina, VP Kannan, Madhu Santhakumar, TV Anupam Kumar

**Affiliations:** 1 Reader, Department of Pedodontics, MES Dental College, Perinthalmanna Kerala, India; 2 Professor and Head, Department of Pedodontics, Government Dental College Thiruvananthapuram, Kerala, India; 3 Professor, Department of Microbiology, Government Medical College Kozhikode, Kerala, India; 4 Professor and Head, Department of Pedodontics, Government Dental College Kozhikode, Kerala, India; 5 Associate Professor, Department of Pedodontics, Government Dental College Kozhikode, Kerala, India; 6 Associate Professor, Department of Pedodontics, Government Dental College Kozhikode, Kerala, India

**Keywords:** Calcium hydroxide, *Candida*, Chlorhexidine, Metronidazole.

## Abstract

**Background:**

*Candida* has been associated with cases of secondary and persistent root canal infections. The purpose of this study was to evaluate and compare the effectiveness of commonly used intracanal medicament against *Candida* biofilms found in root canals of human primary teeth with necrotic pulp.

**Materials and methods:**

Pulp canals of 45 single-rooted primary maxillary anterior teeth with pulp necrosis in 34 children were included in the study. They were divided into three groups of 15 samples each - group I: Ca(OH)_2_ (calcium hydroxide); group II: 1% chlorhexidine gel (CHX); and group III: 1% metronidazole gel. Bacterial count was obtained from each tooth at two different stages: (1) after instrumentation, and (2) after placement of the medication. Statistical analysis using the Statistical Package for the Social Sciences version 10.0 software program (Inc., Chicago, IL, USA) with Wilcoxon signed rank test after grouping the samples was performed.

**Results:**

Ca(OH)_2_, 1% CHX gel, and 1% metronidazole gel were ineffective in completely eliminating *Candida* biofilms from root canal of human primary teeth with necrotic pulp.

**Conclusion:**

None of the commonly used intracanal medicaments, i.e., Ca(OH)_2_, 1% CHX gel, and 1% metronidazole gel, was effective in completely eliminating *Candida* biofilm from root canal of human primary teeth with necrotic pulp. Ineffectiveness of these medicaments against *Candida* has opened new door of research regarding the use of suitable intracanal medicaments against single and multispecies biofilms.

**How to cite this article:**

Paikkatt JV, Sreedharan S, Philomina B, Kannan VP, Santhakumar M, Kumar TVA. Efficacy of Various Intracanal Medicaments in Human Primary Teeth with Necrotic Pulp against *Candida* Biofilms: An *in vivo* Study. Int J Clin Pediatr Dent 2017;10(1):45-48.

## INTRODUCTION

The presence of fungi in the root canals was first reported by Grossman,^1^ who found fungal evidence in 17% of evaluated sample. Fungi have been isolated from root canals of teeth with pulpal necrosis and apical periodontitis^2,3^ and have been reported as a potential cause of endodontic failure in root-filled teeth.^4,5^

*Candida* is the most commonly isolated yeast genus from infected root canal and is potentially pathogenic.^5^ The mechanisms believed to be involved in pathogenesis are (a) adaptability to a variety of environmental conditions; (b) adhesion to a variety of surfaces; (c) production of hydrolytic enzymes; (d) morphologic transition; (e) biofilm formation; and (f) evasion and immunomodu-lation of host defence.^6^

Hence, the elimination of *Candida* from root canals of infected teeth leads to successful treatment.

Endodontic instrumentation cannot effectively eliminate the microflora from root canals of primary teeth mechanically owing to their anatomic complexity and need of patient cooperation.^7^ Hence, dependence on intra-canal medicament for endodontic success becomes more important. Several medicaments have been attempted in root canals of permanent teeth with their respective advantages/disadvantages; however, there is a paucity of literature related to efficacy of various medication used in primary teeth.

Calcium hydroxide [Ca(OH)_2_], is the “gold standard” endodontic medicament used widely to eliminate microbes that survive instrumentation.^8,9^ A 1% chlorhexidine (CHX) gel is an alternate medicament used in endo-dontic practice as an effective antimicrobial means for disinfecting the root canal.^9,10^ Medication was selected in gel form as they have both antimicrobial and lubricating actions during instrumentation.^10^

Because of increasing interest regarding the role of *Candida* biofilms in root canal infections and its association with failed endodontic therapy and recent reports of fungal resistance against some medicaments, the purpose of this clinical study was to find the antimicrobial efficacy of commonly used intracanal medicaments against *Candida* biofilms in root canals of human primary teeth with necrotic pulp.

## MATERIALS AND METHODS

Eligible participants for this clinical trial were selected from patients of both genders aged 4 to 6 years that had been referred for dental treatment at Pediatric Dentistry clinic.

Fulfillment of following inclusion criteria was required for patient enrollment, based on clinical and radiographic examination—asymptomatic maxillary incisor teeth (both central or laterals) with confirmed pulpal necrosis owing to caries (with or without periapi-cal lesion), but with sufficient coronal structure to permit isolation of operative field with rubber dam, less than two-thirds of root resorption, presence or not of fistula, mobility degree 0 or 1, and no periodontal pocket. If present, the periapical lesion should not be invading the follicles of germ of permanent successor. The 34 patients, who met all of these inclusion criteria, were enrolled, providing a total sample of 45 teeth. The study purposes were fully explained to parents/guardians, who signed a written informed consent form. The research protocol was received and approved by institutional Research Ethics Committee.

### Medicaments Tested

- Ca(OH)_2_ paste (RC Cal, Prime Dental Product, India)
- 1% CHX gel (Hexigel, ICPA Ltd., India)
- 1 % metronidazole gel (Metogyl DG gel, Unique Pharmaceuticals, India).

### Methods of the Study

For every appointment, three teeth were selected from patients who met the inclusion criteria. The three maxillary incisors were divided into three experimental groups as described below:

1. Group I—Ca(OH)_2_ paste
2. Group H—1% CHX gel
3. Group III—1% metronidazole gel

After isolation with a rubber dam, the teeth were disinfected with 30% hydrogen peroxide and 10% tincture iodine.^11^ The pulp chambers were opened using aseptic conditions. Airotor with sterile cooled water and highspeed diamond round burs were used. After confirming the working length, instrumentation was performed 1 mm above the apices with Hedstroem (H) files, upto size 50. After the mechanical preparation and irrigation, a sterile, size 20 paper point was introduced into the length of the root canals for the initial microbiological sampling. The paper point was placed for 60 seconds in the canal and then immediately transferred to a sterile test tube. The test tubes containing paper point sample were taken to the Department of Microbiology for processing within 1 hour.^12^

After collecting the sample, the root canals were dried and medicaments placed depending on the group. All medicaments were applied with a syringe and a 26-gauge needle. Subsequently, a sterile cotton pellet was placed at the entrance and the cavities were temporarily sealed with zinc oxide eugenol cement.

For microbiological procedures, the paper point samples were rolled in blood agar plates, and the plate was incubated in an incubator for 48 hours at 37°C.

*Candida* was seen as a nonhemolytic dry white colony; the colony morphology was studied and count of *Candida* was determined as colony-forming unit (CFU) depending on growth on number of streaks. The growth of *Candida* was further confirmed by Gram staining. *Candida* was seen as oval budding yeast 2 to 6 μm in diameter. In order to evaluate the effect of The medicaments against *Candida,* the patients were recalled after 2 weeks.^7^ After irrigation with saline, a second microbiological sampling was carried out with sterile paper points in the root canals as described earlier.

After the microbiological sampling, all the root canals were filled with zinc oxide eugenol paste and all treated teeth were given fiber-reinforced post and crown.

Statistical analysis was carried out using the Statistical Package for the Social Sciences version 10.0 software program (Inc., Chicago, Illinois, USA) with Wilcoxon signed rank test after grouping the samples.

## RESULTS

A total of 45 teeth were studied. They included three groups of 15 teeth each. Group I was medicated with Ca(OH)_2_, group II with 1% CHX gel, and group III with 1% metronidazole gel.

Table 1 outlines that Ca(OH)_2_, 1% CHX gel, and 1% metronidazole gel have no statistically significant action on *Candida.*

**Graph 1:**
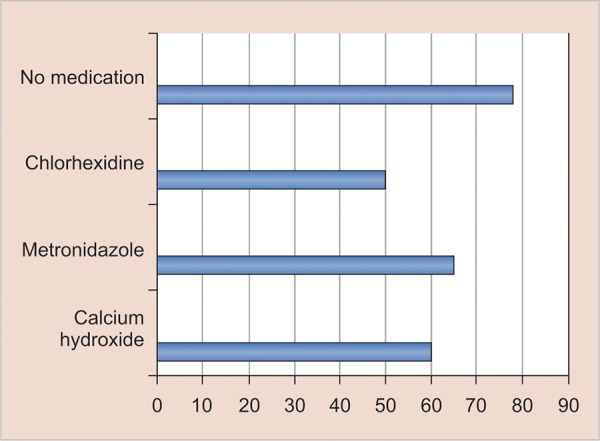
Average count of *Candida* in CFU after various medicaments

After taking the average count of *Candida* in CFUs after various medications, Graph 1 shows that none of the commonly used medicaments, i.e., Ca (OH)_2_, 1% CHX gel, or 1% metronidazole gel, was able to completely eradicate *Candida* from the root canal system.

**Table 1:** **Table 1:** Effect of various medicaments on *Candida*

		*Candida after Ca(OH)_2_ medication* * vs Candida after BMP*		*Candida after CHX vs* * Candida after BMP*		*Candida after metronidazole* * vs Candida after BMP*	
Z		–1.089		–0.577		–1.633	
^b^Asymp. sig. (two-tailed)		0.276		0.564		0.102	
		NS		NS		NS	

^b^Wilcoxon signed rank test; NS: Nonsignificant; BMP: Biomechanical preparation

## DISCUSSION

The antimicrobial effectiveness of 1% CHX gel, Ca(OH)_2_, and 1% metronidazole gel is well investigated *in vitro.* It appeared that they have wide antimicrobial activity against microorganisms isolated from root canal system. However, the results found from *in vitro* study cannot be directly extrapolated to clinical situations because of optimum contact between medication and dentin under *in vitro* condition and because of variable imposed by clinical situation and not reproduced under experimental model. Hence, the present clinical study was undertaken after taking into consideration the above objective.

*Candida* in nature rarely exists in planktonic state, but is organized in biofilm structures, which is a complex community, composed of great variety of organisms with different ecological requirement and pathogenic potential. Biofilm growth is a continuous process that goes through various stages from young to mature, structurally complex biofilm.^13^ In necrotic pulp with harsh ecological milieu, it is likely that the physiological state of biofilm is closest to mature, hence making it resistant to various antimicrobial agents. The protective mechanism underlying biofilm antimicrobial resistance is not fully understood, although several mechanisms have been proposed.^14^ These mechanisms include physical or chemical diffusion barrier to antimicrobial penetration into the biofilm, slow growth of biofilm owing to nutrient limitation, activation of general stress response, and emergence of biofilm-specific phenotype.^15^

The CHX is a cationic bisguanide with broad antimicrobial activity, low mammalian toxicity, and strong affinity to binding to dentin and mucous membrane. The CHX molecules react with negatively charged groups on the cell surface, causing an irreversible loss of cytoplasmic constituents, membrane damage, and enzyme inhibition. It is likely that ionic interaction occurs between the positively charged CHX molecules and negatively charged extracellular matrix. This ionic interaction is understood to reduce the diffusion of fluorescent probes within biofilms by about 50 fold, thus explaining the possible mechanism of *Candida* resistance to 1% CHX gel.^16^

As seen in previous studies, *Candida* was resistant to Ca(OH)_2_. *Candida* survives in a wide range of pH values; the alkalinity of Ca(OH)_2_ may not have any effect on *Candida.* In addition, Ca(OH)_2_ readily displaces Ca^++^ ion necessary for growth and morphogenesis of *Candida.^8,9^*

Metronidazole is bactericidal against most anaerobes that contain electron transport components, such as ferredoxin, which donates electrons to metronidazole, forming highly reactive nitroradical anions that kill susceptible organisms by a radical-mediated mechanism. A 1 % metronidazole gel in this study has not been effective against *Candida,* as it is active against strict anaerobes, but is ineffective against facultative anaerobes.^17,18^

## CONCLUSION

On the basis of observations made during the course of the present study, the following conclusions were drawn.

Firstly, Ca(OH)_2_, 1% CHX gel, and 1% metronidazole gel, which are commonly used intracanal medicaments, were found to be ineffective in completely eliminating *Candida* biofilms from root canals of primary teeth with necrotic pulp. Secondly, ineffectiveness of these medicaments against *Candida* has opened new door of research regarding the use of intracanal medicaments on single and multispecies biofilms.
